# Admission prevalence of colonization with third-generation cephalosporin-resistant *Enterobacteriaceae* and subsequent infection rates in a German university hospital

**DOI:** 10.1371/journal.pone.0201548

**Published:** 2018-08-01

**Authors:** Anne-Cathérine Boldt, Frank Schwab, Anna M. Rohde, Axel Kola, Minh Trang Bui, Nayana Märtin, Marina Kipnis, Christin Schröder, Rasmus Leistner, Miriam Wiese-Posselt, Janine Zweigner, Petra Gastmeier, Luisa A. Denkel

**Affiliations:** 1 Institute of Hygiene and Environmental Medicine, Charité – Universitätsmedizin Berlin, corporate member of Freie Universität Berlin, Humboldt-Universität zu Berlin and Berlin Institute of Health, Berlin, Germany; 2 German Center for Infection Research (DZIF), Braunschweig, Germany; 3 Department of Infection Control and Hospital Hygiene, University Hospital Cologne, Cologne, Germany; Wadsworth Center, UNITED STATES

## Abstract

**Background:**

Many patients admitted to a hospital are already colonized with multi-drug resistant organisms (MDRO) including third-generation cephalosporin-resistant *Enterobacteriaceae* (3GCREB). The aim of our study was to determine the prevalence of rectal 3GCREB colonization at admission to a large German university hospital and to estimate infection incidences. In addition, risk factors for 3GCREB colonization were identified.

**Materials/Methods:**

In 2014 and 2015, patients were screened for rectal colonization with 3GCREB and filled out a questionnaire on potential risk factors at admission to a non-intensive care unit (non-ICU). All patients were retrospectively monitored for bacterial infections. Descriptive, univariable and multivariable logistic regression analyses were conducted to identify risk factors for 3GCREB colonization at admission.

**Results:**

Of 4,013 patients included, 10.3% (n = 415) were rectally colonized with 3GCREB at admission. Incidence of nosocomial infections was 3.5 (95% CI 2.0–6.1) per 100 patients rectally colonized with 3GCREB compared to 2.3 (95% CI 1.8–3.0, P = 0.213) per 100 3GCREB negative patients.

Independent risk factors for 3GCREB colonization were prior colonization / infection with MDRO (OR 2.30, 95% CI 1.59–3.32), prior antimicrobial treatment (OR 1.97, 95% CI 1.59–2.45), male sex (OR 1.38, 95% CI 1.12–1.70), prior travelling outside Europe (OR 2.39, 95% CI 1.77–3.22) and places of residence in the Berlin districts Charlottenburg-Wilmersdorf (OR 1.52, 95% CI 1.06–2.18), Friedrichshain-Kreuzberg (OR 2.32, 95% CI 1.44–3.74) and Mitte (OR 1.73, 95% CI 1.26–2.36).

**Conclusions:**

Admission prevalence of rectal colonization with 3GCREB was high, while infection incidence did not significantly differ between patients rectally colonized or not with 3GCREB at hospital admission. In consequence, hospitals should prioritize improvement of standard precautions including hand hygiene to prevent infections among all patients irrespective of their 3GCREB status at hospital admission.

## Introduction

The burden of third-generation cephalosporin-resistant *Enterobacteriaceae* (3GCREB) is increasing worldwide [1–3]. Antimicrobial resistance is primarily facilitated by the production of extended-spectrum beta lactamases (ESBL). Currently, about 7% of the population of Germany is colonized with ESBL producing *Enterobacteriaceae* (ESBL-E) [4,5]. ESBL enzymes can disrupt a large variety of beta-lactam antibiotics including third-generation cephalosporins (3GC). Recently, Hamprecht et al. reported that 9.5% of patients admitted to German tertiary care hospitals were colonized with 3GCREB [6]. Of those, ESBL production could be determined in more than 90% [6].

Risk factors for colonization with ESBL-E or 3GCREB can be either healthcare- or community-associated. Known healthcare-associated risk factors are prior antimicrobial treatment, previous hospitalization [4,6,7], a stay in a long-term care facility (LTCF), previous colonization with multi-drug resistant organisms (MDRO) and medical treatment of gastroesophageal reflux disease (GERD) [6].

One of the most important community-associated (CA) risk factors is travelling to high prevalence regions including South-East Asia [4,7]. Nutritional habits, including meat consumption (pork, chicken, beef), were described as probable sources of ESBL-E or ESBL-carrying plasmids [8–11]. Thus far, regional or cultural risk factors for 3GCREB and ESBL-E colonization remain poorly understood.

ESBL-E colonizing the human gut have the potential for causing infections [3]. The impact of infections with ESBL-E is controversial. Some studies have reported an association of ESBL-E infections with increased hospital costs, lengths of stay (LOS) and mortality [12,13], while others have not [14,15]. However, inappropriate initial antibiotic treatment has been shown to be more frequently in patients infected with ESBL-E [14,16]. Delayed initiation of adequate antibiotic treatment may lead to increased morbidity and mortality, especially in vulnerable populations, e.g. intensive care unit (ICU) patients [17]. ESBL-E colonization is associated with infection incidence of 4% to 20% with respect to patient population, geography and species analyzed [18–22].

The aim of our study was to analyze the prevalence of rectal 3GCREB colonization at admission to a large German university hospital and to estimate the rate of infections among those with and without rectal 3GCREB colonization. Infections among 3GCREB colonized patients were analyzed in more detail. The secondary objective was to identify possible healthcare- and community-associated risk factors for 3GCREB colonization.

## Materials and methods

### Participants and setting

The study was conducted as part of the multi-center Antibiotic Therapy Optimization Study (ATHOS) [6]. Our report is based on admission screenings at a German university hospital with more than 3,000 beds.

Patients with an age of ≥ 18 years from general wards (anesthesiology, cardiology, dental and oral medicine, gastroenterology, general surgery, gynecology, interdisciplinary unit, hematology / oncology, nephrology, neurology, neurosurgery, orthopedics, radiation therapy, transplant surgery, trauma surgery, urology, vascular surgery) were included in the study. Excluded wards were intensive care units (ICUs), dermatology, obstetrics, ophthalmology, otorhinolaryngology and psychiatry due to expected medical or personal probability to give informed consent to participate in this study. ICU and wards of psychiatry have a high rate of patients not being able to give informed consent for participation in a study, while patients of dermatological, obstetrical, ophthalmological and otorhinolaryngological wards were expected to have a low acceptance of a rectal admission screening. Patients were recruited between May and September 2014 and between April and September 2015, respectively.

Enrolled patients were sampled for colonization with 3GCREB by rectal swabs within 3 days (day 1–3, day of admission = day 1) of admission. Rectal swabs were taken by the patient or the healthcare staff. Each patient was asked to complete a questionnaire regarding potential risk factors for colonization with MDRO including sex, age, current antibiotic treatment, animal contact and previous colonization with MDRO (methicillin-resistant *Staphylococcus aureus* (MRSA), 3GCREB, carbapenem-resistant *Enterobacteriaceae* and vancomycin-resistant enterococci (VRE)). In addition, it inquired about potential risk factors during the 6 months prior to admission: previous antibiotic therapy, travel abroad, stay at rehabilitation center, stay at a long-term care facility (LTCF), hospitalization (in Germany or abroad) and use of antacids or proton-pump inhibitors for gastroesophageal reflux disease (GERD). The questionnaire used in our study can be found in S1 File. In addition, the following information was extracted from electronic patient files: place of residence (Berlin district), nationality classified by World Health Organization (WHO) region [23], and ward of admission. For all patients, presence or acquisition of an infection at admission or during the current hospital stay was analyzed.

### Microbiological methods

Screening swabs (soaked with Amies transport medium) were taken from the rectum and cultivated on ChromID ESBL agar (bioMérieux, Nürtingen, Germany) selecting for ESBL-E. Species identification and antibiotic susceptibility testing of bacteria grown on ChromID ESBL agar was performed by Vitek 2 GN ID and AST N223 card (bioMérieux), respectively. Isolates were included in the study, if they tested non-susceptible to cefotaxime, ceftriaxone or ceftazidime using EUCAST breakpoints [24].

The combination disc test following EUCAST guidelines using cefotaxime, ceftazidime and cefepime with and without clavulanate (Mast Diagnostica, Reinfeld, Germany) was performed to confirm ESBL production [24].

Genotyping of 3GCREB isolates was conducted by repetitive-sequence-based PCR and subsequent microfluidics electrophoresis using the DiversiLab system (bioMérieux).

### Definition of infection

All patients were screened for the presence of bacteria in a clinical specimen (e.g. urine, blood, wound). Electronic patient files of patients tested positive for bacteria in a clinical specimen were examined in order to identify infections present at admission or acquired during the current hospital stay.

Two independent infection control specialists searched patient files for evidence of bacterial infections. A third infection control specialist was consulted in controversial cases. To qualify an infection needed to meet the following criteria: 1) presence of bacteria in a clinical specimen (e.g. urine, blood or wound) and 2) documentation of infection in patient file or appropriate antimicrobial therapy instituted by treating physician. In case of several infections, the first episode was counted.

Infections among patients colonized with 3GCREB were categorized as i) infection with rectal 3GCREB or ii) infection with other bacteria (not rectal 3GCREB). Infection with rectal 3GCREB (i) was determined, if species identification and antibiotic susceptibility testing of rectal and clinical isolates and / or strain-typing analysis were identical. Similarity of antibiotic susceptibility testing was defined by variation of ≤ one two-fold dilution step of minimal inhibitory concentration (MIC). Differences of more than one two-fold dilution step were accepted for single substances, if this was most likely due to selection of resistance by antibiotic exposure. When possible, additional strain typing analysis of rectal and clinical 3GCREB isolates was conducted by repetitive-sequence-based PCR and subsequent microfluidics electrophoresis using the DiversiLab system (bioMérieux).

Community-acquired and nosocomial infections were monitored. All infections acquired within 3 days (day 1–3, day of admission = day 1) of admission were defined as community-acquired. Infections were considered nosocomial, if the patient had been admitted > 3 days earlier than onset of infection.

### Statistical analysis

The prevalence rate of 3GCREB at admission was defined as the number of patients positive for 3GCREB per 100 patients screened. Infection incidence was defined as the number of patients testing positive for infection per 100 patients with LOS > 3 days. Wilson score confidence intervals of 3GCREB prevalence rates and infection incidences (infections / 100 patients) were calculated using Open Source Epidemiologic Statistics for Public Health, V3.01, http://www.openepi.com [25].

In the descriptive analysis, numbers and percentages were calculated. Differences were identified using the Chi-squared or Fisher’s exact test, respectively.

In the multivariable analysis, logistic regression models were applied to identify independent risk factors for colonization with 3GCREB at admission. The following patient-based parameters were considered in the analyses: age (≤45, 46–55, 56–65, 66–75 or > 75); sex (male/ female); prior MDRO colonization; current antibiotic use, antibiotic use during the previous 6 months; travel abroad during the previous 6 months inside or outside Europe; stay at a rehabilitation centre or LTCF during the previous 6 months; hospital stay during the previous 6 months in Germany, in a European country outside Germany or outside Europe; occupational or private animal contact; and treatment of GERD with antacids or proton-pump inhibitors during the previous 6 months. Parameters were categorized as “no” (reference), “yes” or “unknown”. Furthermore, the parameter ward of admission (cardiology, dental and oral medicine, gastroenterology, general surgery, gynecology, hematology / oncology, interdisciplinary unit, neurology, neurosurgery, orthopedics, radiation therapy, transplant surgery, trauma surgery, vascular surgery, other) was included in the analyses. Due to low patient counts, anesthesiology, urology and nephrology were merged into the category “other” wards.

The following parameters identified by the electronic patient files were included: place of residence (not Berlin, Charlottenburg-Wilmersdorf, Friedrichshain-Kreuzberg, Lichtenberg, Marzahn-Hellersdorf, Mitte, Neukölln, Pankow, Reinickendorf, Steglitz-Zehlendorf, Spandau, Tempelhof-Schöneberg, Treptow-Köpenick, unknown) and nationality classified by WHO regions (African region, Region of Americas, South-East Asia Region, European Region, Eastern Mediterranean Region and Western Pacific Region, unknown) [23]. The variables “wards of admission”, “place of residence” and “region of origin” were dummy-coded. Reference categories for these variables were all other “wards of admission”, “places of residence” or “regions of origin”, respectively.

In the multivariable analysis, the model building strategy was performed stepwise backward, the significance level for excluding a parameter from the model was p = 0.05. For epidemiological reasons, age and sex were included in all models. P values < 0.05 were considered significant. All analyzes were performed using SPSS 22 (IBM SPSS Statistics, Somer, NY, USA) and SAS 9.3 (SAS Institute, Cary, NC, USA).

## Results

### 3GCREB colonization at admission to the hospital

**Fig 1:**
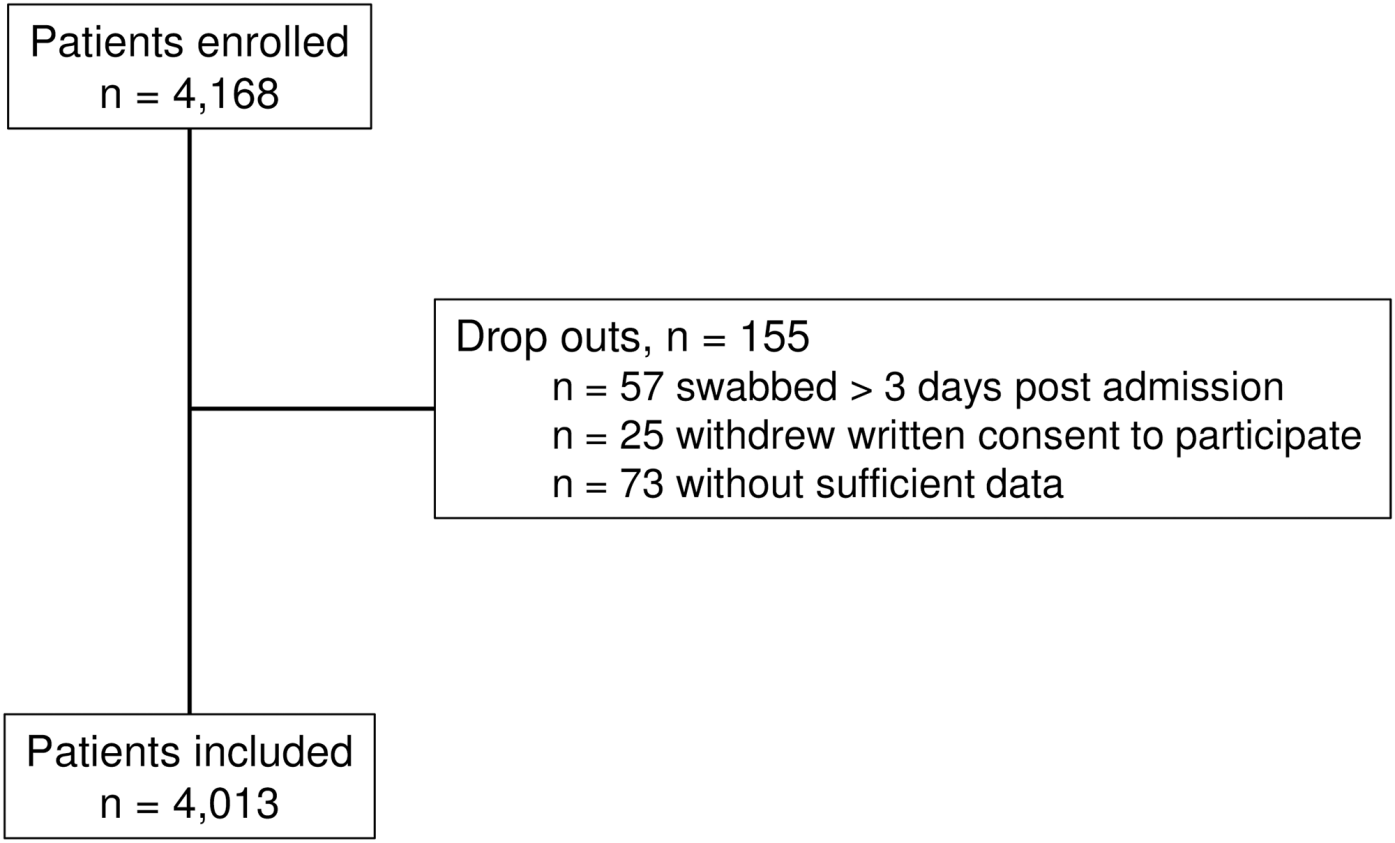
Flow diagram for study participants included in the 3GCREB prevalence study, Berlin, Germany, 2014/2015.

Overall, 4,013 patients were included in this prevalence study. A flow diagram for study participants is shown in Fig 1. Median age of the patients was 62 years (inter quartile range (IQR) 50–73), 50.3% (n = 2,019) were female. Charlson comorbidity index (CCI) was available for 97.1% (n = 3,900) of patients. Median CCI did not differ between all patients (CCI 3, IQR 1–5), 3GCREB negative patients (CCI 3, IQR 1–5) and 3GCREB colonized patients (CCI 3, IQR 1–6). Prevalence of 3GCREB colonization at admission was 10.3% (415 of 4,013 patients, 95% CI 9.4–11.3%).

### Microbiology of 3GCREB isolates

Fourteen patients (0.3%) were colonized with two different 3GCREB strains. Microbiological analysis of 429 3GCREB isolates from 415 patients is summarized in Table 1. Of 429 *Enterobacteriaceae* isolated, 264 (61.5%) were resistant only to 3GC, 160 (37.3%) 3GCREB isolates were also resistant to fluoroquinolones (FQ), and 5 isolates (1.2%) carried resistance to 3GC, FQ and carbapenemes (C) simultaneously. The species most frequently identified among 3GCREB isolates were *Escherichia (E*.*) coli* (82.1%, 352 of 429 isolates), followed by *Klebsiella (K*.*) pneumoniae* (7.9%, 34 isolates), *Enterobacter* spp. (5.6%, 24 isolates), *Citrobacter* spp. (3.0%, 13 isolates), *Klebsiella oxytoca* (0.9%, four isolates) and *Hafnia alvei* (0.5%, two isolates). Extended-spectrum beta-lactamase (ESBL) production was detected in 392 of 429 3GCREB isolates (91.4%).

**Table 1:** Distribution of resistances among 429 3GCREB isolates from 415 patients, 3GCREB prevalence study, Berlin, Germany, 2014/2015.

		Total	*E*.*coli*, n (%)	*Klebsiella pneumoniae*, n (%)	*Enterobacter* spp., n (%)	*Citrobacter* spp., n (%)	*Klebsiella oxytoca*, n (%)	*Hafnia alvei*, n (%)
**Total**		429 (100%)	352 (82.1%)	34 (7.9%)	24 (5.6%)	13 (3.0%)	4 (0.9%)	2 (0.5%)
	ESBL	392 (91.4%)	345 (80.4%)	34 (7.9%)	7 (1.6%)	3 (0.7%)	2 (0.5%)	1 (0.2%)
	No ESBL	37 (8.6%)	7 (1.6%)	0 (0.0%)	17 (4.0%)	10 (2.3%)	2 (0.5%)	1 (0.2%)
**Resistant to 3GC**		264 (61.5%)	215 (61.1%)	14 (41.2%)	20 (83.3%)	11 (84.6%)	3 (75%)	1 (50%)
	ESBL	234 (54.5%)	213 (60.5%)	14 (10.4%)	4 (16.7%)	1 (7.7%)	2 (50%)	0 (0.0%)
	No ESBL	30 (7.0%)	2 (0.6%)	0 (0.0%)	16 (66.7%)	10 (76.9%)	1 (25%)	1 (50%)
**Resistant to 3GC + FQ**		160 (37.3%)	135 (38.4%)	20 (58.9%)	3 (12.5%)	1 (1.7%)	0 (0.0%)	1 (50%)
	ESBL	155 (36.1%)	131 (37.2%)	20 (58.9%)	2 (8.3%)	1 (1.7%)	0 (0.0%)	1 (50%)
	No ESBL	5 (1.2%)	4 (1.4%)	0 (0.0%)	1 (4.2%)	0 (0.0%)	0 (0.0%)	0 (0.0%)
**Resistant to 3GC + FQ + C**		5 (1.2%)	2 (0.6%)	0 (0.0%)	1 (4.2%)	1 (1.7%)	0 (0.0%)	0 (0.0%)
	ESBL	3 (0.7%)	1 (0.3%)	0 (0.0%)	1 (4.2%)	1 (1.7%)	0 (0.0%)	0 (0.0%)
	No ESBL	1 (0.2%)	1 (0.3%)	0 (0.0%)	0 (0.0%)	0 (0.0%)	0 (0.0%)	0 (0.0%)

3GC—third generation cephalosporins, FQ—fluorquinolones, C—carbapenemes

### Infections

**Fig 2:**
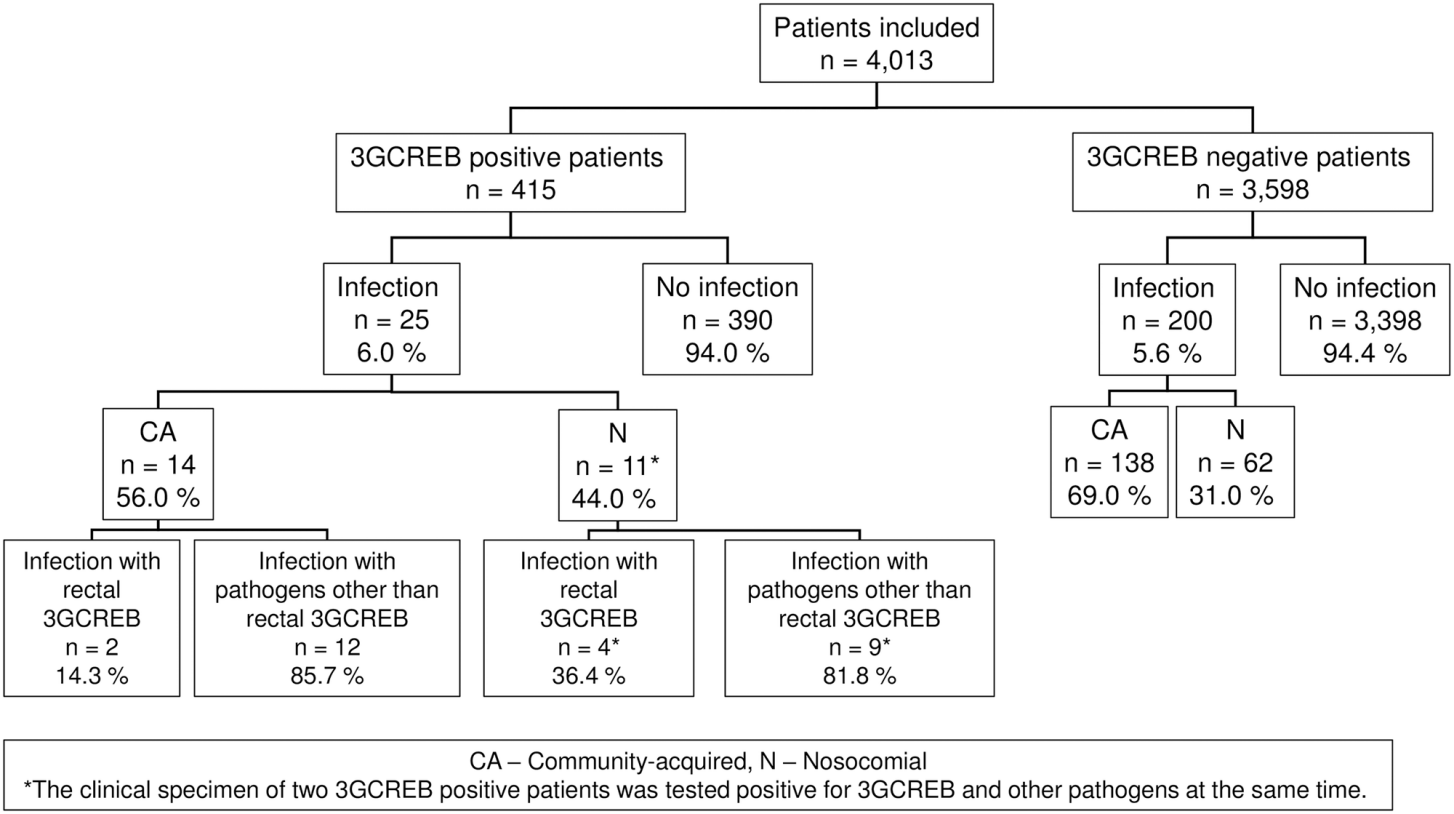
Overview of infections among study participants, 3GCREB prevalence study, Berlin, Germany, 2014/2015. CA—community-acquired, N—nosocomial. Asterisks indicate that the clinical specimen of two patients colonized with 3GCREB were tested positive for 3GCREB and other pathogens at the same time.

225 (5.6%) patients with infection were identified among 4,013 study participants. An overview of infections among all patients stratified by 3GCREB colonization status at hospital admission can be found in Fig 2. Incidences of nosocomial infections were calculated for patients with LOS > 3 days screened for colonization with 3GCREB at hospital admission (Table 2). Median time until onset of nosocomial infection was 10.5 days (6–17 days) among all patients, 9 days (IQR 6–18.5 days) among 3GCREB colonized patients and 10.5 days (6–16 days) among patients not colonized with 3GCREB at hospital admission.

**Table 2:** Infection incidences (with 95%CI) per 100 patients among 2,931 patients screened for 3GCREB colonization at admission to the hospital and with LOS > 3 days. Patients stratified by positive (n = 316) or negative 3GCREB status (n = 2,615) at hospital admission, 3GCREB prevalence study, Berlin, Germany, 2014/2015.

Parameter	3GCREB status at admission	3GCREB status at admission	P value
	**Negative**	**Positive**	
**All infections**	2.3 (1.8–3.0)	3.5 (2.0–6.1)	0.213
**Infections with rectal 3GCREB**		1.3 (0.5–3.2)	
**Infections with other pathogens (not rectal 3GCREB)**		2.9 (1.5–5.3)	

95% CI—Confidence interval. P values were calculated by Chi-Squared test.

#### Infections among 3GCREB negative patients

Infections diagnosed among 3GCREB negative patients were infected wounds (n = 62, 30.9%), urinary tract infections (UTI, n = 55, 27.4%), bloodstream infections (BSI, n = 38, 18.9%), pneumonia (n = 18, 9.0%), intra-abdominal infections (n = 11, 5.5%), *Clostridium difficile* infections (CDI, n = 7, 3.5%), urosepsis (n = 5, 2.5%), and others (n = 5, 2.5%). The latter included enteritis caused by *Salmonella infantis* or *Campylobacter jejuni*, bacterial abscesses and an infected shoulder joint. One patient was diagnosed with pneumonia and UTI at the same time. Pathogens identified most frequently as causative agents of infections were: *Escherichia coli* (n = 55, 27.3%), *Staphylococcus aureus* (n = 29, 14.4%), coagulase-negative staphylococci (n = 25, 12.4%), *Enterococcus* spp. (n = 19, 9.5%) and *Streptococcus* spp. (n = 16, 8.0%).

#### Infections among 3GCREB colonized patients

Among the 415 3GCREB positive patients, 25 (6.0%) had an infection at admission or developed an infection during the current hospital stay. Six (1.4%) of 415 patients suffered from infections with the rectal 3GCREB and 21 (5.1%) with bacterial pathogens other than the rectal 3GCREB. Two of these patients (0.5%) had an infection caused by 3GCREB and other pathogens simultaneously (Fig 2). Pathogens other than 3GCREB detected most frequently in infected patients were *E*.*coli* susceptible to 3GC, *Pseudomonas aeruginosa*, *Enterococcus faecalis*, vancomycin resistant enterococci (VRE), *Staphylococcus aureus* and *Klebsiella pneumoniae* susceptible to 3GC (S1 Table).

Of 25 patients with an incident infection, 11 patients (44.0%) acquired infection(s) during the current hospital stay (> 3 days post admission), four of them with 3GCREB, nine with other pathogens. Two of these patients acquired infections with 3GCREB and other pathogens simultaneously (Fig 2). Median time until onset of nosocomial infection among 3GCREB colonized patients was 6 days (IQR 4.75–10.5 days) for infections with rectal 3GCREB and 12 days (IQR, 6.0–20.0 days) for infections with other pathogens.

The infection diagnosed most frequently among 3GCREB positive patients was urinary tract infection (UTI, 12 of 25 patients; 48.0%), followed by bloodstream infections (BSI, 6 of 25 patients; 24.0%), intra-abdominal infections (6 of 25 patients, 24.0%) and one infected wound (4.0%).

Of 352 patients colonized with 3GCR-*E*.*coli*, 4 (1.1%) developed an infection (two UTIs, one intra-abdominal infection, one BSI) with 3GCR-*E*.*coli*. However, in 34 patients colonized with 3GCR-*Klebsiella pneumoniae*, this agent accounted for one BSI and one intra-abdominal infection (2 of 34 patients, 5.9%). This difference (1.0% versus 5.9%, P = 0.181) was not determined to be significant. Two of six 3GCREB colonized patients with subsequent 3GCREB infection in this study were co-infected with VRE *faecium* (S1 Table).

Similarity of rectal and clinical isolates was tested by comparing the results of species identification and antibiotic susceptibility testing. Antimicrobial susceptibility of rectal isolates and the respective clinical isolates were determined to be identical in six patients. In two of these patients rectal isolates and the respective clinical specimen were available for strain-typing analysis. This molecular analysis showed that the clinical specimen (urine, blood) of both 3GCREB infections tested were identical with the respective rectal isolate (S1 Fig). An overview of all 3GCREB positive patients with infections at admission or during the current hospital stay is shown in S1 Table.

### Risk factor analysis for 3GCREB colonization

**Fig 3:**
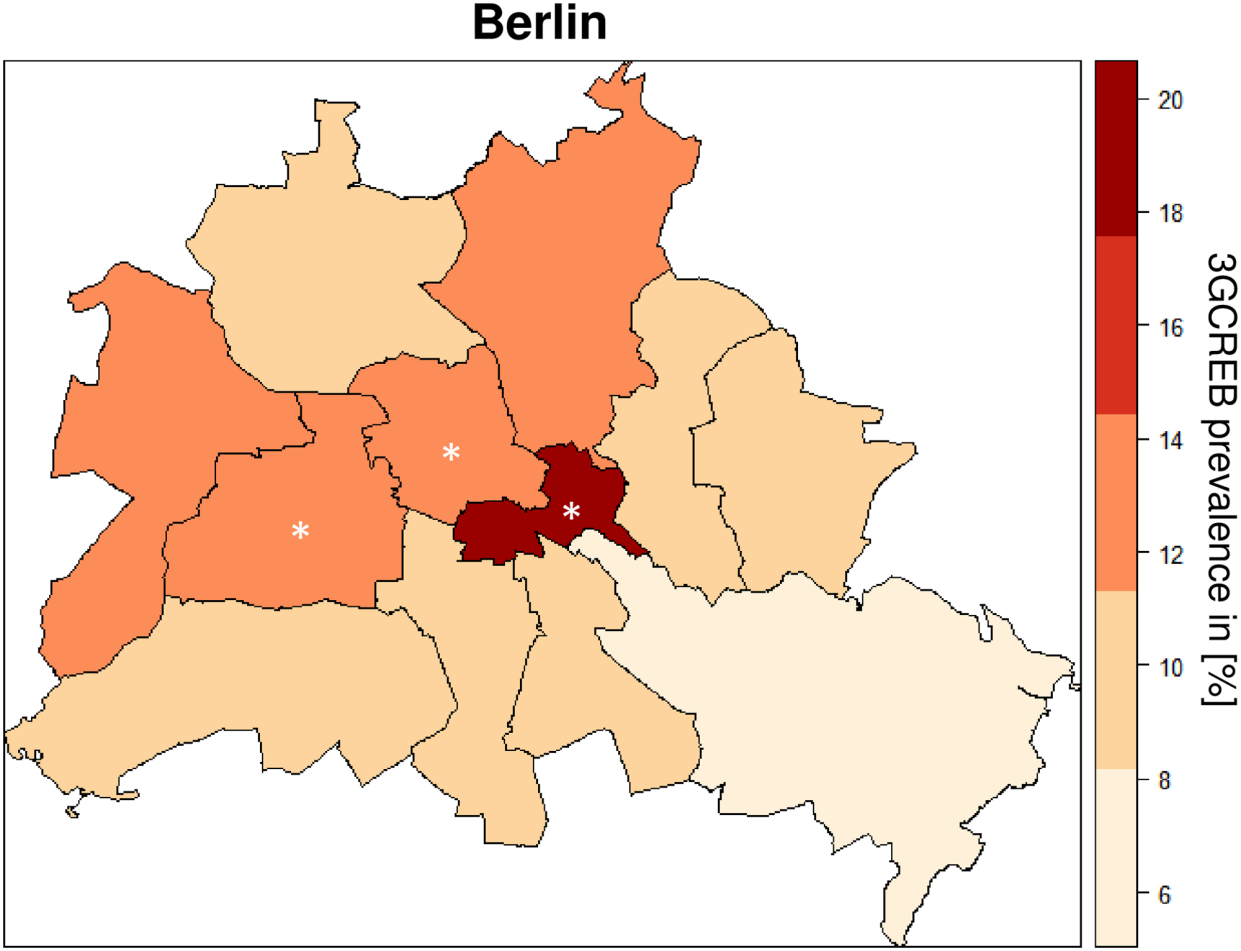
Comparison of Berlin district-dependent prevalence (in %) of rectal colonization with 3GCREB at admission to the hospital. Asterisks indicate districts with significantly increased 3GCREB prevalence compared to other districts (multivariable logistic regression analysis). The map was adjusted according to the geodata reference map published by a German newspaper [26]. 3GCREB prevalence study, Berlin, Germany, 2014/2015.

The complete descriptive analysis is presented in Table 3 and S2 Table. In brief, it shows that 3GCREB colonized patients were significantly more often male (56.9% versus 49.2%, P = 0.003), took antibiotics at the time of admission (21.4% versus 15.5%, P = 0.002), and had been previously colonized or infected with MDRO (10.1% versus 4.5%, P<0.001). Furthermore, patients more frequently tested positive for 3GCREB if—with respect to the previous 6 months—they had taken antibiotics (3.9% versus 2.9%, P<0.001), travelled outside Europe (16.1% versus 7.5%, P<0.001), been admitted to a German hospital (38.3% versus 31.6%, P = 0.012), stayed in LTCF (9.2% versus 8%, P = 0.024) or were being treated for GERD with antacids or proton-pump inhibitors (43.6% versus 38.6%, P = 0.048). Patients colonized with 3GCREB at hospital admission lived significantly more often in Friedrichshain-Kreuzberg (5.8% versus 2.7%, P < 0.001) and Mitte (13.7 versus 9.7, P = 0.010) and less frequently outside Berlin (18.8 versus 24.3, P = 0.012). 3GCREB prevalence in Berlin stratified by district is shown in Fig 3 [26].

**Table 3:** Descriptive ysis of demographic patient data of 4,013 patients screened for 3GCREB colonization at admission to the hospital. Patients stratified by positive or negative 3GCREB status at admission, 3GCREB prevalence study, Berlin, Germany, 2014/anal 2015.

		3GCREB status at admission		Prevalence per 100 patients	P-value
Parameter	Category	Negative	Positive	Positive	
**Patient**		3598 (100%)	415 (100%)	10.3	
**Sex**	**Male**	1770 (49.2%)	236 (56.9%)	11.8	0.003*
**Age [years]**	**≤ 45**	689 (19.1%)	83 (20.0%)	10.8	0.854
	**46–55**	639 (17.8%)	65 (15.7%)	9.2	
	**56–65**	743 (20.7%)	84 (20.2%)	10.2	
	**66–75**	895 (24.9%)	107 (25.8%)	10.7	
	**> 75**	632 (17.6%)	76 (18.3%)	10.7	
Ward of admission*	**Cardiology**	666 (18.5%)	62 (14.9%)	8.5	0.074
	**Dental and oral medicine**	237 (6.6%)	31 (7.5%)	11.6	0.495
	**Gastroenterology**	603 (16.8%)	80 (19.3%)	11.7	0.196
	**General surgery**	81 (2.3%)	10 (2.4%)	11.0	0.837
	**Gynecology**	104 (2.9%)	14 (3.4%)	11.9	0.581
	**Hematology/oncology**	159 (4.4%)	27 (6.5%)	14.5	0.056
	**Interdisciplinary unit**	71 (2%)	10 (2.4%)	12.3	0.550
	**Neurology**	331 (9.2%)	32 (7.7%)	8.8	0.317
	**Neurosurgery**	150 (4.2%)	18 (4.3%)	10.7	0.871
	**Orthopedics**	309 (8.6%)	43 (10.4%)	12.2	0.227
	**Radiation therapy**	37 (1%)	3 (0.7%)	7.5	0.553
	**Transplant surgery**	35 (1%)	8 (1.9%)	18.6	0.074
	**Trauma surgery**	599 (16.6%)	51 (12.3%)	7.8	0.022
	**Vascular surgery**	211 (5.9%)	25 (6%)	10.6	0.896
	**Other**	5 (0.1%)	1 (0.2%)	16.7	0.611
Place of residence (Berlin district)*	**Charlottenburg-Wilmersdorf**	257 (7.1%)	40 (9.6%)	13.5	0.066
	**Friedrichshain-Kreuzberg**	98 (2.7%)	24 (5.8%)	19.7	0.001
	**Lichtenberg**	81 (2.3%)	8 (1.9%)	9.0	0.672
	**Marzahn-Hellersdorf**	86 (2.4%)	9 (2.2%)	9.5	0.779
	**Mitte**	350 (9.7%)	57 (13.7%)	14.0	0.010
	**Neukölln**	176 (4.9%)	18 (4.3%)	9.3	0.618
	**NotBerlin**	876 (24.3%)	78 (18.8%)	8.2	0.012
	**Pankow**	159 (4.4%)	24 (5.8%)	13.1	0.207
	**Reinickendorf**	216 (6%)	22 (5.3%)	9.2	0.566
	**Spandau**	94 (2.6%)	12 (2.9%)	11.3	0.737
	**Steglitz-Zehlendorf**	643 (17.9%)	67 (16.1%)	9.4	0.383
	**Tempelhof-Schöneberg**	427 (11.9%)	47 (11.3%)	9.9	0.746
	**Treptow-Köpenick**	125 (3.5%)	8 (1.9%)	6.0	0.096
	**Unknown**	10 (0.3%)	1 (0.2%)	9.1	> 0.999
Region of origin*	**African region**	2 (0.1%)	0 (0.0%)	0.0	
	**Eastern Mediterranean Region**	9 (0.3%)	3 (0.7%)	25.0	0.095
	**European region**	2458 (68.3%)	278 (67.0%)	10.2	0.582
	**Region of Americas**	7 (0.2%)	0 (0.0%)	0.0	
	**South-East Asian Region**	1 (0.0%)	0 (0.0%)	0.0	
	**Western Pacific region**	2 (0.1%)	0 (0.0%)	0.0	
	**Unknown**	1119 (31.1%)	134 (32.3%)	10.7	0.621

P-values were calculated by Chi-Squared test or Fisher’s exact test, respectively.

*P-values ≤ 0.05 were considered significant.

The parameters place of residence, ward of admission and region of origin were dummy-coded.

The category “Other” in ward of admission includes anesthesiology, nephrology and urology.

Independent risk factors for 3GCREB colonization at hospital admission according to the final multivariable model were prior MDRO colonization / infection (OR = 2.30, 95% CI = 1.59–3.32), antimicrobial treatment (OR = 1.97, 95% CI = 1.59–2.45) and travelling outside Europe (OR = 2.39, 95% CI = 1.77–3.22) during the previous 6 months. Further risk factors were male sex (OR = 1.38, 95% CI = 1.12–1.70), places of residence in Charlottenburg-Wilmersdorf (OR = 1.52, 95% CI = 1.06–2.18), Mitte (OR = 1.73, 95% CI = 1.26–2.36) and Friedrichshain-Kreuzberg (OR = 2.32, 95% CI = 1.44–3.74). Protective factors associated with a reduced risk of 3GCREB colonization were admission to a cardiology ward (OR = 0.73, 95% CI = 0.55–0.98) or a trauma surgery ward (OR = 0.67, 95% CI = 0.48–0.91). The multivariable analysis is summarized in Table 4.

**Table 4:** Results of the multivariable conditional logistic regression analysis of 4,013 patients to identify risk factors for colonization with 3GCREB at admission, 3GCREB prevalence study, Berlin, Germany, 2014/2015.

Parameter	Category	Odds Ratio	95% confidence interval	P-value
**Sex**	Male	1.38	1.12–1.70	0.003
**Previous MDRO colonization / infection**	Yes	2.30	1.59–3.32	0.001
**Antibiotic use during the previous 6 months**	Yes	1.97	1.59–2.45	0.005
**Travelling to a non-European country during the previous 6 months**	Yes	2.39	1.77–3.22	0.028
**Place of residence**	Charlottenburg-Wilmersdorf	1.52	1.06–2.18	0.024
	Friedrichshain-Kreuzberg	2.32	1.44–3.74	0.001
	Mitte	1.73	1.26–2.36	0.001
**Ward of admission**	Cardiology	0.73	0.55–0.98	0.037
	Trauma surgery	0.67	0.48–0.91	0.012

P-values ≤ 0.05 were considered significant.

## Discussion

Prevalence of 3GCREB colonization at hospital admission was high, while infection incidence did not significantly differ between patients positive or negative for rectal colonization with 3GCREB at hospital admission. Further, we identified that community-associated risk factors including travelling outside Europe and living in certain urban areas might play an important role in 3GCREB colonization at hospital admission. In consequence, for non-ICU patients, effectiveness of cost and labor intense measures including general admission screenings to prevent transmission of 3GCREB colonization within the hospital may be questioned. Instead, hospitals should focus on improvement of standard precautions including hand hygiene to prevent infections among all patients irrespective of their 3GCREB colonization status at hospital admission.

### 3GCREB colonization at admission to the hospital

The 3GCREB prevalence of 10.3% identified in this study is similar to recently published findings demonstrating that 9.5% of patients were tested positive for 3GCREB at admission to German hospitals [6]. Other studies investigating the prevalence of ESBL-E and ESBL-producing *E*. *coli* in the population of Germany reported lower prevalence rates of 6 to 7% [4,5]. [1–3]. Hamprecht et al. and our study examined patients at admission to the hospital but not the general population. The difference between these populations is illustrated by the high percentage of patients included in our study reporting use of antibiotics during the previous six months (>30%). This might explain the higher 3GCREB prevalence in patients admitted to the hospital. Furthermore, the range of 3GCREB prevalence in Germany varied between 5.1% and 11.8% depending on the hospital of admission [6]. Thus, regional differences are likely to have an impact. Antibiotic resistance was facilitated by ESBL production in more than 90% of our 3GCREB isolates; more than 80% of those isolates were *E*. *coli*. This makes a comparison of our data with studies investigating ESBL-E and ESBL-*E*. *coli* possible.

### Infections

Interestingly, infection incidence among patients colonized with 3GCREB was not significantly higher compared to patients not colonized with 3GCREB at hospital admission. In fact, incidence of infections with the colonizing 3GCREB was very low among 3GCREB positive patients at hospital admission. Carriers of ESBL-E were shown to have varying rates of subsequent infections depending on patient population, geography and the type of infection analyzed. A French hospital had ESBL-E infection incidence of 8% [19]. The rate differed between 4% and 20% among ESBL-E carriers in two French ICUs [20,22]. Furthermore, 8.5% of ESBL-E colonized patients in American ICUs developed ESBL-E-BSI [21], while in an Israeli hospital 15.4% of patients with fecal ceftazidime-resistant *Enterobacteriaceae* colonization had a subsequent bacteremia with the same species [18]. The low incidence of infections with the rectal 3GCREB among patients colonized with 3GCREB at hospital admission observed in the present study might be explained by the fact that we focused on patients from general wards and not high-risk patients. Below 5% of patients were admitted to a hematological / oncological ward, while ICU patients were not considered in this analysis. Recently, high-risk patients identified by a score-assigned prediction model were shown to have a significantly higher cumulative probability of developing an infection with multi-drug resistant Gram-negative bacteria (MDRGN) than lower risk patients [27].

The most frequent infections with the rectal 3GCREB were UTI, BSI and intra-abdominal infections, reflecting the gastrointestinal and urinary tract as typical colonization sites of ESBL-E [28]. Even though the majority of 3GCREB infections (all BSI, all intra-abdominal infections) in our study were nosocomial, we also detected 3GCREB-UTIs not acquired during the current hospital stay. Thus, infections with 3GCREB, especially UTI, are not restricted to the hospital [29,30].

In this study, nosocomial infection was defined by onset of infection > 3 days post admission. Due to this strict definition, incidence of nosocomial infections might be underestimated. However, we performed a sensitivity analysis. Infection incidences did not significantly differ, if onset of infections ≥ 3 days post admission were considered nosocomial (2.5 infections per 100 patients with onset > 3 days post admission vs. 2.3 infections per 100 patients with onset ≥ 3 days, P = 0.624).

### Risk factors for 3GCREB colonization at hospital admission

Known health care-associated risk factors for ESBL-E colonization are antibiotic treatment and prior colonization or infection with MDRO [6,31–36]. These risk factors were also detected in our study. Moreover, as previously shown by others, male gender could be associated with 3GCREB colonization [18,30,35].

Multivariable analysis identified admission to trauma surgery and cardiology wards as independent protective factors for 3GCREB colonization. This finding might be explained by the facts that patients admitted to the ward of trauma surgery usually do not have a history of previous hospitalization and in most cases have fewer secondary diseases. Patients admitted to the ward of cardiology usually have a lower rate of previous antibiotic consumption.

In addition to healthcare-associated risk factors, the present study also focused on community-associated risk factors for 3GCREB colonization. Travel outside Europe is reported as one of the most important risk factors for ESBL-E colonization and was also identified by our study [4,7]. In particular, contact with the Middle East / South Asia (MESA) has a significant association with ESBL-E colonization [4,7,11,37]. The multivariable logistic regression analysis identified residence in Friedrichshain-Kreuzberg, Mitte and Charlottenburg-Wilmersdorf as independent risk factors for 3GCREB colonization at admission. We can only speculate as to reasons for these regional differences. Interestingly, Friedrichshain-Kreuzberg, Mitte and Charlottenburg-Wilmersdorf are the only Berlin districts without a border to Berlin’s city limits. These three districts also have the highest residential and traffic densities per hectar [38]. Transmission might be more likely in urban areas with more frequent exposures to 3GCREB, e.g. in households, apartment buildings, public transport, or supermarkets. A Spearman rank order correlation found a strong correlation between 3GCREB prevalence and population density (r = 0.62, P = 0.033) [38]. In contrast, no correlation was identified for households of ≥ 4 person (r = 0.36, P = 0.245) or for foreigners from the Eastern Mediterranean region (r = 0.22, P = 0.484) living in Berlin districts [38]. However, a causal relationship between ESBL colonization and population density cannot be concluded from our data. ESBL transmission is complex and not yet fully understood, especially regarding community-associated risk factors including cultural and nutritional habits [3]. Recently, living in Parisean area was identified with an elevated risk of ESBL-E colonization [39]. Certain urban areas might represent a surrogate parameter for the complexity of risk factors for ESBL-E colonization. Such a combination of risk factors might include crowded housing conditions or frequent contact to high prevalence areas, not only by nationality or travel, but also by having visitors or consuming food from those areas. Having an Asian native language or a full name whose origin is in MESA were previously reported as further surrogate parameters for the complexity of ESBL transmission [11,37].

### Strengths and limitations

Our study has several limitations. First, this is a monocenter analysis done in a university hospital. The ability to draw any general conclusions for other (tertiary care) hospitals is limited. However, median CCI in our cohort is 3 (IQR 1–5), while median CCI among other non-ICU patient cohorts in German university hospitals were reported to vary between 2 and 5.6 depending on underlying diseases [11,40,41]. In consequence, we expect our patient cohort to be comparable to other non-ICU patients in German university hospitals.

Second, despite careful examination of infections among 3GCREB positive patients in electronic patient files independently by two infection control specialists, infections might have been missed due to insufficient reporting by treating physicians or not taking enough cultures. This would lead to an underestimation of infection incidence.

Third, similarity of rectal and clinical 3GCREB isolates was tested by comparing antibiotic susceptibility testing and not by molecular analyses [42]. Unfortunately, rectal and clinical isolates were available for only two of six 3GCREB colonized patients with 3GCREB infection. However, similarity of rectal and clinical isolates could be verified by repetitive PCR-based typing method in both cases. In consequence, our low 3GCREB infection rate might be overestimated.

Fourth, two of six 3GCREB positive patients with subsequent 3GCREB infection were co-infected with VRE *faecium*. The fact that patients are increasingly co-infected with more than one multi-drug resistant organism including methicillin-resistant *Staphyloccocus aureus* (MRSA), VRE, and ESBL-E has been shown previously [43]. Thus, the causing agent of these infections cannot be clearly identified. In this study, those infections were counted as both, 3GCREB infections and infections with other pathogens. In consequence, this might lead to an overestimation of 3GCREB infection rate. However, the overestimation of 3GCREB infection rate does not change the conclusion of our study. Fifth, no discharge surveillance was done. If patients were discharged before onset of infection, this infection was missed. Sixth, the definition of nosocomial infections in this study refers to the current hospital stay. We cannot exclude that community-acquired infections might have been acquired during a previous stay in the hospital or another healthcare institution.

Strengths of our study were the high number of patients included and the fact that our study was done during the same season (May—September) on the same wards within two consecutive years (2014 and 2015). Further, this study included all species of 3GCREB and did not focus on *E*.*coli* alone or excluded ESBL negative *Enterobacteriaceae*. We performed one of the most extensive analyses of risk factors for colonization with 3GCREB including healthcare- and community-associated parameters. To our knowledge, this is the first prevalence study calculating infection incidences for non-ICU patients stratified by the 3GCREB colonization status at hospital admission.

### Outlook

The epidemiology of 3GCREB colonization is still not fully understood, especially in the field of community-associated risk factors. Further studies including molecular analysis of 3GCREB isolates, e.g. by whole genome sequencing, are necessary to understand the epidemiology and sources of these widespread multi-drug resistant Gram-negative organisms.

## Supporting information

S1 Table Microbiological overview of all 25 3GCREB colonized patients with infections at admission or during the current hospital stay. Strain typing was done for patient 3* and patient 5*, 3GCREB prevalence study, Berlin, Germany, 2014/2015.
(DOCX)

S2 Table Descriptive analysis of information received by the questionnaire answered by 4,013 patients screened for 3GCREB colonization at admission to the hospital. Patients stratified by positive or negative 3GCREB status at admission, 3GCREB prevalence study, Berlin, Germany, 2014/2015. P-values were calculated by Chi-Squared test or Fisher’s exact test, respectively. P-values ≤ 0.05 were considered significant. * ^1^at the time of answering the questionnaire.
(DOCX)

S1 Fig Strain typing analysis for comparison of rectal admission screening swabs with clinical swabs of patient 3 and patient 5. 1: 3GCR + FQR-*Escherichia coli* from rectal admission screening swab of patient 3, 2: 3GCR + FQR-*Escherichia coli* from blood of patient 3, 3: 3GCR-*Escherichia coli* from rectal admission screening swab of patient 5, 4: 3GCR-*Escherichia coli* from urine of patient 5. 3GCR—resistant to third-generation cephalosporins, FQR—resistant to fluorquinolones.
(TIF)

S1 File Case report form of ATHOS prevalence study with questionnaire on risk factors for colonization with MDRO. (DOCX)
