# Supplementary material for: Admission prevalence of colonization with third-generation cephalosporin-resistant Enterobacteriaceae and subsequent infection rates in a German university hospital
Source: PLoS One. 2018 Aug 1;13(8):e0201548. doi: 10.1371/journal.pone.0201548 (PMC6070276; doi:10.1371/journal.pone.0201548)
Supplement: S1 Table — Strain typing was done for patient 3* and patient 5*, 3GCREB prevalence study, Berlin, Germany, 2014/2015. (DOCX) [file pone.0201548.s001.docx]

| **Patient** | **Colonizing 3GCRE** | **Type of infection** | **Pathogens identified in clinical specimen** | **Category of infection (3GCREB / Other)** | **Nosocomial (N) / Community-associated (CA)** | **Time to onset of infection (days)** |
| --- | --- | --- | --- | --- | --- | --- |
| **1** | 3GCR+ FQR-*E.coli* | Intra-abdominal infection | 3GCR + FQR-*E.coli*, 3GC + FQR – Klebsiella pneumoniae, Vancomycin-resistant *Enterococcus faecium* | 3GCREB, other | N | 21 |
| **2** | 3GCR-*Klebsiella pneumoniae* | Intra-abdominal infection | 3GC-*Klebsiella pneumoniae , Morganella morganii*, *Citrobacter brakii,* Vancomycin-resistant *Enterococcus faecium* (VRE), *Enterococcus faecium* | 3GCREB, other | N | 5 |
| **3*** | 3GCR-*E.coli* | UTI | 3GCR-*E.coli* | 3GCREB | N | 4 |
| **4** | 3GCR +-*E.coli* | UTI | 3GCR + FQR-*E.coli* | 3GCREB | CA | 1 |
| **5*** | 3GCR + FQR-*E.coli* | BSI | 3GCR + FQR-*E.coli* | 3GCREB | N | 7 |
| **6** | 3GCR + FQR-*K. pneumoniae* | BSI | 3GCR + FQR-*K. pneumoniae* | 3GCREB | CA | 2 |
| **7** | 3GCR + FQR-*E.coli* | UTI | 3GCR-*E.coli* | Other | N | 6 |
| **8** | 3GCR + FQR-*K. pneumoniae* | UTI | *Enterococcus faecalis* | Other | CA | 1 |
| **9** | 3GCR + FQR-*E.coli* | UTI | *E.coli* | Other | CA | 3 |
| **10** | 3GCR-*E.coli* | UTI | *E.coli* | Other | CA | 1 |
| **11** | 3GCR + FQR-*E.coli* | UTI | *E.coli* | Other | CA | 2 |
| **12** | 3GCR + FQR-*E.coli* | Intra-abdominal infection | *Streptococcus mitis* / *oralis*, *Pediococcus pentosaceus* | Other | N | 12 |
| **13** | 3GCR + FQR-*E.coli* | UTI | *E.coli, Klebsiella oxytoca* | Other | CA | 2 |
| **14** | 3GCR + FQR-*E.coli* | BSI | *Staphylococcus aureus* | Other | N | 17 |
| **15** | 3GCR-*Citrobacter freundii* | UTI | *Klebsiella pneumoniae* | Other | CA | 2 |
| **16** | 3GCR-*E.coli* | Urosepsis | *E.coli* | Other | CA | 1 |
| **17** | 3GCR-*E.coli* | Intra-abdominal infection | *Klebsiella pneumoniae* | Other | CA | 2 |
| **18** | 3GCR-*Enterobacter spp.* | Intra-abdominal infection | *Pseudomonas aeruginosa*, *Streptococcus mitis / oralis*, 3GCR + FQR-*Pseudomonas aeruginosa* | Other | CA | 3 |
| **19** | 3GCR-*E.coli* | Infected wound | *Staphylococcus aureus, Pseudomonas aeruginosa* | Other | CA | 2 |
| **20** | 3GCR + FQR-*E.coli* | BSI | *Staphylococcus haemolyticus* | Other | N | 24 |
| **21** | 3GCR-*E.coli* | UTI | *Streptococcus agalacticae*, *Pseudomonas aeruginosa*, *Enterococcus faecalis* | Other | N | 9 |
| **22** | 3GCR-*E.coli* | BSI | *Staphylococcus hominis* | Other | N | 20 |
| **23** | 3GCR-*E.coli* | Intra-abdominal infection | *Klebsiella pneumoniae*, *Streptococcus anginosus*, *Staphylococcus aureus* | Other | N | 6 |
| **24** | 3GCR-*Enterobacter cloacae complex* | UTI | *Enterobacter cloacae complex* | Other | CA | 2 |
| **25** | 3GCR + FQR-*E.coli* | BSI | *Enterococcus faecalis* | Other | CA | 3 |

P-values were calculated by Chi-Squared test or Fisher’s exact test, respectively. P-values ≤ 0.05 were considered significant. * ^1^at the time of answering the questionnaire. 3GCR- resistant to third generation cephalosporins, FQR – resistant to fluorquinolones
