# Supplementary material for: Admission prevalence of colonization with third-generation cephalosporin-resistant Enterobacteriaceae and subsequent infection rates in a German university hospital
Source: PLoS One. 2018 Aug 1;13(8):e0201548. doi: 10.1371/journal.pone.0201548 (PMC6070276; doi:10.1371/journal.pone.0201548)
Supplement: S2 Table — Patients stratified by positive or negative 3GCREB status at admission, 3GCREB prevalence study, Berlin, Germany, 2014/2015. P-values were calculated by Chi-Squared test or Fisher’s exact test, respectively. P-values ≤ 0.05 were considered significant. * 1at the time of answering the questionnaire. (DOCX) [file pone.0201548.s002.docx]

|  |  | **3GCREB status at admission** |  | **Prevalence per 100 patients** | **P-value** |
| --- | --- | --- | --- | --- | --- |
| **Parameter** | **Category** | **Negative** | **Positive** | **Positive** |  |
| **Patient** |  | 3598 (100%) | 415 (100%) | 10.3 |  |
| **Current^1^ antibiotic use** | Unknown | 621 (17.3%) | 80 (19.3%) | 11.4 | 0.002* |
|  | Yes | 556 (15.5%) | 89 (21.4%) | 13.8 |  |
|  | No | 2421 (67.3%) | 246 (59.3%) | 9.2 |  |
| **Previous MDRO colonization / infection** | Unknown | 119 (3.3%) | 17 (4.1%) | 12.5 | < 0.001* |
|  | Yes | 162 (4.5%) | 42 (10.1%) | 20.6 |  |
|  | No | 3317 (92.2%) | 356 (85.8%) | 9.7 |  |
| **Antibiotic use during the previous 6 months)** | Unknown | 106 (2.9%) | 16 (3.9%) | 13.1 | < 0.001* |
|  | Yes | 1053 (29.3%) | 188 (45.3%) | 15.1 |  |
|  | No | 2439 (67.8%) | 211 (50.8%) | 8.0 |  |
| **Travelling in Europe (during the previous 6 months)** | Unknown | 27 (0.8%) | 5 (1.2%) | 15.6 | 0.401 |
|  | Yes | 568 (15.8%) | 58 (14%) | 9.3 |  |
|  | No | 3003 (83.5%) | 352 (84.8%) | 10.5 |  |
| **Travelling outside Europe (during the previous 6 months)** | Unknown | 27 (0.8%) | 5 (1.2%) | 15.6 | < 0.001* |
|  | Yes | 269 (7.5%) | 67 (16.1%) | 19.9 |  |
|  | No | 3302 (91.8%) | 343 (82.7%) | 9.4 |  |
| **Stay in rehabilitation center (during the previous 6 months)** | Unknown | 4 (0.1%) | 2 (0.5%) | 33.3 | 0.168 |
|  | Yes | 294 (8.2%) | 36 (8.7%) | 10.9 |  |
|  | No | 3300 (91.7%) | 377 (90.8%) | 10.3 |  |
| **Stay in LTCF (during the previous 6 months)** | Unknown | 2 (0.1%) | 2 (0.5%) | 50.0 | 0.024* |
|  | Yes | 288 (8%) | 38 (9.2%) | 11.7 |  |
|  | No | 3308 (91.9%) | 375 (90.4%) | 10.2 |  |
| **Hospital stay in Germany (during the previous 6 months)** | Unknown | 50 (1.4%) | 8 (1.9%) | 13.8 | 0.012* |
|  | Yes | 1138 (31.6%) | 159 (38.3%) | 12.3 |  |
|  | No | 2410 (67%) | 248 (59.8%) | 9.3 |  |
| **Hospital stay in Europe (during the previous 6 months)** | Unknown | 50 (1.4%) | 8 (1.9%) | 13.8 | 0.185 |
|  | Yes | 268 (7.4%) | 40 (9.6%) | 13.0 |  |
|  | No | 3280 (91.2%) | 367 (88.4%) | 10.1 |  |
| **Hospital stay outside Europe (during the previous 6 months)** | Unknown | 50 (1.4%) | 8 (1.9%) | 13.8 | 0.671 |
|  | Yes | 7 (0.2%) | 1 (0.9%) | 13.0 |  |
|  | No | 3541 (98.4%) | 406 (97.8%) | 10.1 |  |
| **Occupational animal contact (during the previous 6 months)** | Unknown | 2 (0.1%) | 0 (0%) | 0.0 | 0.783 |
|  | Yes | 1188 (33%) | 132 (31.8%) | 10.0 |  |
|  | No | 2408 (66.9%) | 283 (68.2%) | 10.5 |  |
| **Private animal contact (during the previous 6 months)** | Unknown | 2 (0.1%) | 1 (0.2%) | 33.3 | 0.334 |
|  | Yes | 46 (1.3%) | 7 (1.7%) | 13.2 |  |
|  | No | 3550 (98.7%) | 407 (98.1%) | 10.3 |  |
| **Treatment of GERD (during the previous 6 months)** | Unknown | 57 (1.6%) | 2 (0.5%) | 3.4 | 0.040* |
|  | Yes | 1389 (38.6%) | 181 (43.6%) | 11.5 |  |
|  | No | 2152 (59.8%) | 232 (56.4%) | 9.6 |  |

P-values were calculated by Chi-Squared test or Fisher’s exact test, respectively. P-values ≤ 0.05 were considered significant (*). ^1^at the time of answering the questionnaire. MDRO – multidrug resistant organisms, LTCF – long term care facility, GERD – gastroesophageal reflux disease.
