# Supplementary figures and images for: Admission prevalence of colonization with third-generation cephalosporin-resistant Enterobacteriaceae and subsequent infection rates in a German university hospital
Source: PLoS One. 2018 Aug 1;13(8):e0201548. doi: 10.1371/journal.pone.0201548 (PMC6070276; doi:10.1371/journal.pone.0201548)

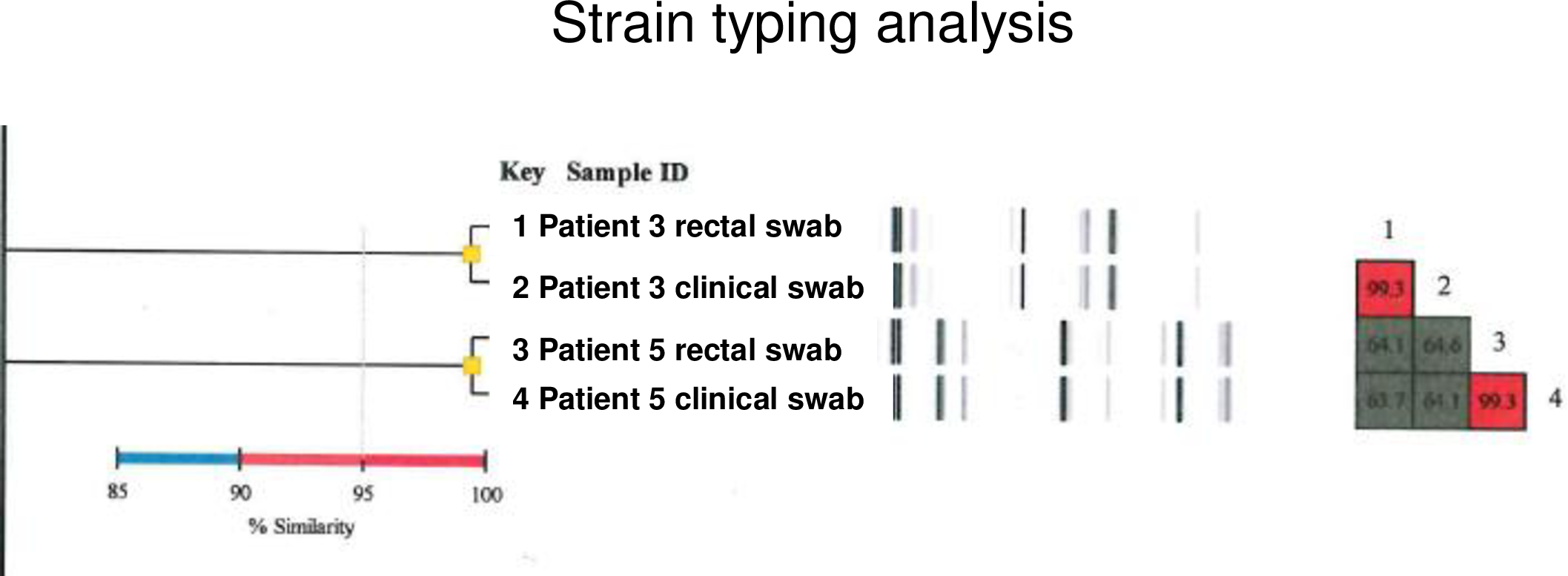

Supplement: S1 Fig — 1: 3GCR + FQR-Escherichia coli from rectal admission screening swab of patient 3, 2: 3GCR + FQR-Escherichia coli from blood of patient 3, 3: 3GCR-Escherichia coli from rectal admission screening swab of patient 5, 4: 3GCR-Escherichia coli from urine of patient 5. 3GCR—resistant to third-generation cephalosporins, FQR—resistant to fluorquinolones. (TIF) [file pone.0201548.s003.tif]
