# Supplementary material for: Admission prevalence of colonization with third-generation cephalosporin-resistant Enterobacteriaceae and subsequent infection rates in a German university hospital
Source: PLoS One. 2018 Aug 1;13(8):e0201548. doi: 10.1371/journal.pone.0201548 (PMC6070276; doi:10.1371/journal.pone.0201548)
Supplement: S1 File — (DOCX) [file pone.0201548.s004.docx]

**Put Pat.ID sticker here**

**Recruiter initials**

**ATHOS prevalence study**

**A. Patient information**

| **Patient- ID (Studynumber)** | | |  | |
| --- | --- | --- | --- | --- |
| Unit-ID |  | Age in years |  | Sex **❒ M ❒  F** |

| **Entry date to ATHOS-Area (DD.MM.YY)** |  |
| --- | --- |
| **Entry date to ATHOS-Unit (DD.MM.YY)** |  |
| **Date of rectal swab (DD.MM.YY)** |  |
| **Current AB therapy (oral/iv)?**  **❒ Yes** ❒** No** ❒** Unknown**  **❒ Ja** ❒** Nein** ❒** Unbekannt**  **❒ Ja** ❒** Nein** ❒** Unbekannt** | |

**B. Results of Screening**

**Negative ❒**

**Positive ❒**

| **Microbiological Finding** ( resistant *Citrobacter*, *Escherichia*, *Enterobacter*, *Klebsiella*, *Salmonella*, *Serratia*, *Proteus* and *Hafnia* species) | | | |
| --- | --- | --- | --- |
| **Isolate 1**  (genus + species) |  | **Isolate 2**  (genus + species) |  |
| **MDR-GN** | **❒3GCREB ❒3MDR-GN ❒4MDR-GN** | **MDR-GN** | **❒3GCREB ❒3MDR-GN ❒4MDR-GN** |
| **VRE** | **❒Yes** ❒**No** | **VRE** | **❒Yes** ❒**No** |
| **ESBL** (omit if VRE) | **❒Positive** ❒**Negative** | **ESBL** (omit if VRE) | **❒Positive** ❒**Negative** |
| **Colony number** | **❒ + ❒ ++ ❒ +++** | **Colony number** | **❒ + ❒ ++ ❒ +++** |

**Risk factor questionnaire**

| 1. **Were you ever diagnosed with a multidrug-resistant organism (colonisation or infection)?**   **❒ Yes** ❒** No** ❒** Unknown**  If yes, which type? **❒**MRSA **❒**VRE **❒**ESBL- Producer **❒**3GCREB **❒**3MDR-GN **❒**4MDR-GN **❒**Unknown   1. **Did you take antibiotics in the previous 6 months (not including a current AB Therapy)?**   **❒ Yes** ❒** No** ❒** Unknown**   1. **Have you been abroad in the last 6 months?**   **❒ Yes** ❒** No** ❒** Unknown**  If yes, up to three entries : **Within Europe name country by hand** and **outside Europe pick a region**:    _________________________ _________________________ _________________________  **❒** Africa **❒**Asia (indicate extra if in **❒**India) **❒**North America **❒** Central and South Amerika **❒**Australia + Oceania **❒**Arabian Peninsula     1. **Have you been to a rehabilitation facility in the last 6 months?**   **❒ Yes** ❒** No** ❒** Unknown**   1. **Have you been to a long-term care facility in the last 6 months?**   **❒ Yes** ❒** No** ❒** Unknown**   1. **Have you been to a hospital in Germany or abroad for stationary care in the last 6 months?**   **❒ Yes** ❒** No** ❒** Unknown**  If yes, up to three entries : **Within Europe name country by hand** and **outside Europe pick a region**:    _________________________ _________________________ _________________________  **❒** Africa **❒**Asia (indicate extra if in **❒**India) **❒**North Amerika **❒** Central and South Amerika **❒**Australia + Oceania **❒**Arabian Peninsula   1. **Do you have occupational animal contact?**   **❒ Yes** ❒** No** ❒** Unknown**   1. **Do you have pets?**   **❒ Yes** ❒** No** ❒** Unknown**   1. **Did you take medication against gastroesophageal reflux disease in the last 6 months?**   **❒ Yes** ❒** No** ❒** Unknown**  Medication against gastroesophageal reflux disease are e.g.:  a. Antacids  b. Proton-pump inhibitors |
| --- |
